# Spin–orbit coupling in buckled monolayer nitrogene

**DOI:** 10.1038/s41598-022-07215-2

**Published:** 2022-02-25

**Authors:** Paulina Jureczko, Marcin Kurpas

**Affiliations:** Institute of Physics, University of Silesia in Katowice, 41-500 Chorzów, Poland

**Keywords:** Spintronics, Two-dimensional materials

## Abstract

Buckled monolayer nitrogene has been recently predicted to be stable above the room temperature. The low atomic number of nitrogen atom suggests, that spin–orbit coupling in nitrogene is weak, similar to graphene or silicene. We employ first principles calculations and perform a systematic study of the intrinsic and extrinsic spin–orbit coupling in this material. We calculate the spin mixing parameter $b^2$, reflecting the strength of the intrinsic spin–orbit coupling and find, that $b^2$ is relatively small, on the order of $10^{-6}$. It also displays a weak anisotropy, opposite for electrons and holes. To study extrinsic effects of spin–orbit coupling we apply a transverse electric field enabling spin–orbit fields $\Omega$. We find, that $\Omega$ are on the order of a single $\mu$eV in the valence band, and tens to a hundred of $\mu$eV in the conduction band, depending on the applied electric field. Similar to $b^2$, $\Omega$ is also anisotropic, in particular for the conduction electrons.

## Introduction

Two-dimensional (2D) pnictogens have gained a lot of attention in recent years^1–10^. Among these materials atomically thin black phosphorus emerged as the most promising one for electronic and spintronic applications due to its intrinsic semiconducting band gap, extraordinary high carrier mobility and strongly anisotropic orbital and spin properties^1,2,11,12^. Recently, 2D atomically thin nitrogen, called also *nitrogene*^13^, has been predicted to be structurally stable even far above the room temperature^10,13,14^. According to these predictions, 2D nitrogene can crystallize in a few different forms, each having distinct electronic properties. Puckered (black phosphorus like) nitrogene is a direct gap semiconductor with the gap 1.25 eV^15^. Octagon nitrogene is an indirect wide-gap semiconductor with the gap 2.6 eV^16,17^, while buckled honeycomb nitrogene is an indirect gap insulator with the calculated band gap of about 4–6 eV^13^. The wide band gap of buckled nitrogene may be an obstacle to use this material in semiconductor electronics. However, several ways of lowering the gap to the semiconducting limit have been proposed, including formation of multilayers^14^, doping by boron^18^ or making nitrogene nanoribbons^13^.

So far the experimental realization of 2D nitrogene remains a challenge. The main obstacle here is a tendency of N atoms to form $\hbox {N}_2$ molecules with a triple N$\equiv$N bond^19^. Therefore, the goal is to find conditions under which this tendency is minimized, allowing for the formation of single bonds between N atoms. Such conditions can be created, for instance, by high pressure and temperature. Very recently this strategy has led to the synthesis of bulk nitrogen in black phosphorus structure^19,20^. More useful method, in terms of practical applications of nitrogene, was used by Harada et al., who epitaxially grown a single atom thick N layer in GaAs^21^. Although the structure of the epitaxial layer did not correspond to any of the theoretically envisaged, this is a big step towards the realization of 2D nitrogene.

**Figure 1:**
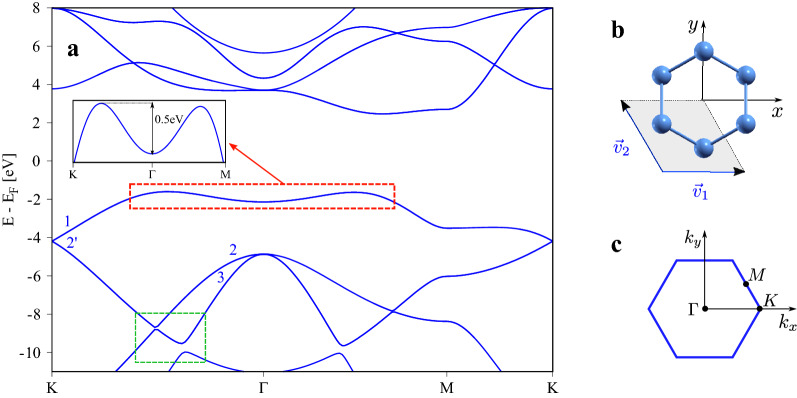
Electronic properties of monolayer nitrogene: (**a**) Calculated relativistic band structure of nitrogene plotted along high symmetry lines of the First Brillouin Zone shown in (**c**). The inset enlarges the valence band around the $\Gamma$ point. The two maxima lie approximately 0.5 eV above the minimum at the $\Gamma$ point; (**b**) crystalline structure of 2D nitrogene with the lattice vectors $\mathbf {v}_1$, $\mathbf {v}_2$, $|\mathbf {v}|=0.69$ Å and the unit cell marked by the shaded area; (**c**) The First Brillouin Zone of 2D nitrogene with depicted high symmetry points.

Nitrogen is a light element with the atomic number Z=14. Therefore, spin–orbit coupling (SOC) in nitrogene is expected to be weak^22^, similar to graphene^23^ and phosphorene^12^. Indeed, in a recent theoretical study, Lee et al.^10^ observed no significant effects of SOC on the band structure of buckled nitrognene. This may open great perspectives for this material in spintronics, since weak SOC should result in long lasting spin coherence. Buckled 2D nitrogene crystallizes in the centrosymmetric structure of the $P3{\bar{m}}1$ space group being isomorphic with the $D_{3d}$ point group. The unit cell of nitrogene contains two nonequivalent atoms (Fig. 1b) belonging to two noncoplanar sublattices A and B. The finite out of plane buckling of the structure breaks the mirror symmetry of the lattice and allows the emergence of the *intrinsic Rashba*^24,25^ spin–orbit coupling (PIA SOC in the context of functionalized graphene^26,27^). In contrast to the well known Bychkov–Rashba^28^ SOC due to structure inversion asymmetry, the intrinsic Rashba SOC preserves the spin degeneracy of states, as a consequence of space inversion symmetry of the lattice and the time reversal symmetry. On the other hand, it affects the electron spin and leads to the emergence of in-plane spin components, forbidden by symmetry in flat, mirror symmetric graphene.

Besides the above general considerations, not much is known about SOC in this material. For instance, the question about the competition of the intrinsic Rashba SOC, polarizing the spins in-plane, and the intrinsic SOC polarizing spins along the *z* directions has not been addressed. Since such a competition may lead to the anisotropy of the SOC, we take up this problem here and perform a systematic study of SOC in buckled monolayer nitrogene. By employing first principles calculations we characterize the intrinsic SOC by the spin mixing parameter $b^2$^29^, while the extrinsic SOC, arising due to breaking of the space inversion symmetry, is quantified by the amplitude of spin splittings $\Delta _{so}$ and spin–orbit fields $\Omega$. All these quantities provide essential information about the strength and anisotropy of SOC in the band structure.

We find, that $b^2$ is of the order of $10^{-6}$, both for electrons and holes, and displays a weak in-plane to out-of plane anisotropy. The extrinsic SOC shows significant diversity between the valence and conduction bands. In the former, the values of spin–orbit fields $\Omega$ vary between a few to a dozen of $\mu$eV for the considered amplitudes of an external electric field, and are rather isotropic. In the latter, the values of $\Omega$ are roughy ten times bigger and are strongly anisotropic.

## Results and discussion

### Intrinsic spin–orbit coupling

We begin our considerations from the examination of the electronic band structure of nitrogene. The calculated first principles relativistic band structure is shown in Fig. 1. Similar to other buckled 2D materials made of group V elements, such as blue phosphorene, arsenene or antimonene^22^, the band gap of monolayer nitrogene is indirect. The size of the indirect gap for the PBE exchange correlation functional is $E_g=4.06$, eV, and is in agreement with the values reported by others^13^. The top-most valence band has two local maxima lying between the $\Gamma$K and $\Gamma$M paths in the First Brillouin Zone (FBZ) (Fig. 1c). The maxima are located approximately 0.5 eV above the saddle point at the $\Gamma$ point, and differ in energy by 35 meV. The edge of the conduction band is located in the middle of the $\Gamma$M path in the FBZ.

As already stated by Lee et al.^10^, the effects of spin–orbit coupling on the band structure on buckled nitrogene are weak. Indeed, the inclusion of relativistic effects in the calculation makes no significant effects on the non-relativistic band structure. The most prominent ones are spin–orbital gaps opened at high symmetry points of the FBZ. In the valence band, the spin–orbital gap at the $\Gamma$ point (between the bands marked 2 and 3) is $\Delta ^{\Gamma }_{SO} = 17.3$ meV, while at the *K* point (between the bands marked 1 and 2’) it is $\Delta ^{K}_{SO} = 2.1$ meV. For comparison, the same splittings for graphene are $\Delta ^{\Gamma }_{SO} = 9$ meV and $\Delta ^{K}_{SO} = 24$ $\mu$eV^23^, and for blue phosphorene $\Delta ^{\Gamma }_{SO} = 48$ meV, $\Delta ^{K}_{SO} = 10$ $\mu$eV^22^.

To characterize the intrinsic SOC in the band structure away from the high symmetry points we calculate the spin mixing parameter $b^2$. This parameter measures the amplitude of the spin component being admixed to the Bloch state of opposite spin by the SOC. Importantly, the parameter $b^2$ can be easily accessed experimentally from the Elliott relation connecting $b^2$ with the spin $\tau _s$ and momentum $\tau _p$ relaxation times: $b^2 = \tau _p ( 4 \tau _s)^{-1}$ , provided the Elliott-Yafet mechanism dominates spin relaxation^29,30^. This is usually the case, when the spin lifetime follows the same characteristics as the momentum lifetime^31–34^. Knowing $\tau _p$ and $\tau _s$ from the experiment, one can extract the sample independent parameter $b^2$, and compare it with the theoretical values. Recently we have successfully applied this strategy to characterize $b^2$ and spin relaxation in black phosphorus^12,34^. In numerical simulations the parameter $b^2$ can be calculated directly from the wave functions, provided the spin subbands of a Bloch state $\psi _{\mathbf {k},n}$ are degenerate. This requirement is met if the time reversal symmetry and space inversion symmetry of the sample are present simultaneously. In such a case the two Bloch subbands are

1 $$\begin{aligned} \psi _{\mathbf {k},n}^{\Uparrow }(\mathbf {r})= & {} [a_{k,n}(\mathbf {r})|\uparrow \,\rangle + b_{k,n}(\mathbf {r})|\downarrow \,\rangle ]e^{i\mathbf {k} \cdot \mathbf {r}}, \end{aligned}$$

2 $$\begin{aligned} \psi _{\mathbf {k},n}^{\Downarrow }(\mathbf {r})= & {} [a^{*}_{-k,n}(\mathbf {r})|\downarrow \,\rangle - b^{*}_{-k,n}(\mathbf {r})|\uparrow \,\rangle ]e^{i\mathbf {k} \cdot \mathbf {r}}, \end{aligned}$$

where *n* is the band index, $a_{k,n}$ and $b_{k,n}$ are lattice periodic functions, $\mathbf {k}$ is the crystal momentum and $|\uparrow \rangle$, $|\downarrow \rangle$ are eigenstates of the spin one-half operator with eigenvalues $\pm \hbar /2$^35^. Here, $b_{k,n}$ is the amplitude of spin component $\vert \sigma ^{'} \rangle$ being admixed by the SOC to the Bloch state of spin $\vert \sigma \rangle$, $\sigma =\lbrace \uparrow ,\downarrow \rbrace$. Since the Bloch states $\psi _{\mathbf {k},n}^{\Uparrow }(\mathbf {r})$ and $\psi _{\mathbf {k},n}^{\Downarrow }(\mathbf {r})$ are degenerate at any *k*-point, any linear combination of these states is also an eigenstate of the Hamiltonian. This allows us to chose the amplitudes $a_{k,n}$ and $b_{k,n}$ in such a way, that $\psi _{\mathbf {k},n}^{\Uparrow }(\mathbf {r})$, $\psi _{\mathbf {k},n}^{\Downarrow }(\mathbf {r})$ diagonalize spin one-half operator $\hat{s_i}$, $i=x,y,z$. By doing so we can choose the spin quantization axis (SQA), SQA=*i*, which in experiment corresponds to the direction of magnetization of injected spins.

The spin mixing parameter can be calculated by integrating the amplitude $|b_{k,n,}|$ over the whole unit cell

3 $$\begin{aligned} {b^{2}_{\mathbf {k},n}} = \int |b_{\mathbf {k},n}(\mathbf {r})|^{2}d^{3}\mathbf {r}, \end{aligned}$$

or, alternatively by calculating the deviation of the expectation value of the spin operator ${\hat{s}}_i$ from its nominal value 1/2 (in units of $\hbar$)^36^

4 $$\begin{aligned} b^{2}_{\mathbf {k},n,i} = \frac{1}{2} -|\langle \psi _{\mathbf {k},n}^{\sigma ,i}(\mathbf {r})| {\hat{s}}_i| \psi _{\mathbf {k},n}^{\sigma ,i}(\mathbf {r})\rangle | , \end{aligned}$$

where $\sigma =\lbrace \Uparrow , \Downarrow \rbrace$ and $\psi _{\mathbf {k},n}^{\sigma ,i}(\mathbf {r})$ is the eigenstate of ${\hat{s}}_i$. For normalized states $0\le b^{2}_{\mathbf {k},n,i}\le 0.5$, where $b^{2}_{\mathbf {k},n,i} = 0.5$ corresponds to fully spin mixed states and $b^{2}_{\mathbf {k},n,i} = 0$ to pure spin up (down) spinors.

**Figure 2:**
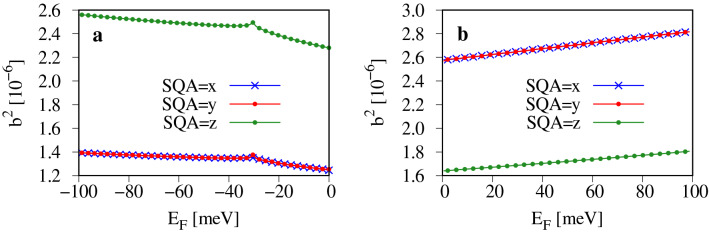
Calculated Fermi contour averaged spin-mixing parameter $b^2$ versus the position of the Fermi level $E_F$ and for three directions of SQA aligned with the Cartesian system axes: (**a**) for the valence band; (**b**) for the conduction band. The Fermi level is measured with respect to the valence (conduction) band maximum (minimum).

The calculated Fermi contour averaged spin mixing parameter $b^2$ plotted versus the position of the Fermi level is shown in Fig. 2. The value of $b^2$ is in the range of $10^{-6}$ for both, the valence and the conduction band. This is roughly ten times the value of $b^2$ for graphene and ten times less than for black and blue phosphorus^22,34,37^. At $E_F \approx 32$ meV the second valence band maximum (along the $\Gamma M$ path in Fig. 1) crosses the Fermi level and *k*-points from this wedge of the FBZ start contributing to $b^2$, slightly modifying its slope. The parameter $b^2$ is almost doping independent. Such behavior is typical for bands being energetically separated from other lower and higher lying bands^22^.

The parameter $b^2$ shows weak anisotropy with respect to the spin quantization axis. In the valence band (Fig. 2a) $b^2$ for SQA=z (out of plane polarization) is roughly twice as large as for SQA=*x*, *y*, $b^2_{SQA=x/y}/b^2_{SQA=z}\approx 0.54$. This result is surprising, since most of buckled elemental monolayer materials display the opposite trend^22^. In this context nitrogene is similar to graphene, which exhibits similar anisotropy of $b^2$ for holes away from the Dirac point. For conduction electrons (Fig. 2b) we observe the opposite trend in $b^2$, namely, $b^2_{SQA=x/y}/b^2_{SQA=z}\approx 1.6$. Although the anisotropy of $b^2$ is not very high, this result deviates from other buckled pnictogens, for which $b^2$ is mostly isotropic.

The results presented Fig. 2 have been obtained for the PBE exchange–correlation functional. Since for hybrid functionals the band gap increases by 2 eV^13^, we have checked how the band gap correction influences the values of the spin mixing parameter and performed calculations for the HSE^38^ functional. Although the band gap increased to 6.34 eV (see Supplementary Fig. S1) the corresponding spin mixing parameter (Supplementary Fig. S2) stays almost unaffected and varies by at most a few percent. Therefore, the results obtained for the PBE functional can be taken as conclusive.

### Extrinsic spin–orbit coupling

Monolayer nitrogene has been predicted to be structurally stable on metal surfaces. Even though the interaction with the substrate is weak and makes no significant changes to the band structure of nitrogene^13^, the crystal potential at the interface breaks the inversion symmetry of the nitrogene lattice and enables the extrinsic Bychkov–Rashba spin–orbit coupling^28^. The extrinsic SOC has two main effects on the electron spin. First, it removes the degeneracy of spin states, and second, it induces crystal momentum dependent spin–orbital fields $\pmb {\Omega }_{k}$, which lead to spin polarization of Bloch states and to the emergence of the characteristic spin texture in the FBZ. The SO field $\pmb {\Omega }_{k}$ is linked to spin splitting by the Zeeman-like Rashba Hamiltonian

5 $$\begin{aligned} H_R( k ) = \frac{\hbar }{2} \pmb {\Omega }_{k} \cdot \pmb {\sigma } , \end{aligned}$$

where $\hbar$ is the Planck constant, and $\varvec{\sigma }$ is the vector of Pauli matrices.

Instead of placing nitrogene on a particular substrate we model its presence by applying an external transverse electric field $\pmb {E}=(0,0,E)$, whose amplitude can be precisely controlled in numerical calculations. This approach, allowing us to simulate different substrates, is justified due to weak hybridization of states of nitrogene and the substrate^13^.

**Figure 3:**
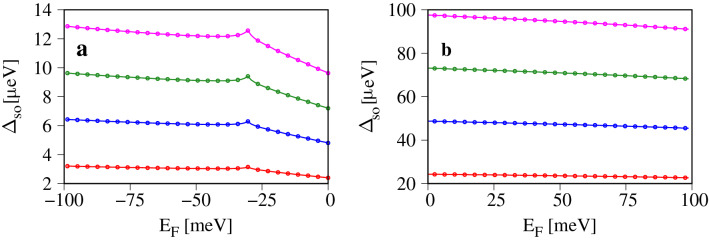
Fermi contour averaged spin splitting versus Fermi level for several values of the external electric field E, and for: (**a**) the top most valence band; (**b**) the bottom most conduction band. The Fermi level is measured with respect to the valence (conduction) band maximum (minimum).

In Fig. 3 we show the Fermi contour averaged spin splitting $\Delta _{so}$ calculated for several values of the electric field. In contrast to the spin mixing parameter $b^2$, $\Delta _{so}$ differs significantly between the valence and conduction band. In the former (Fig. 3a), $\Delta _{so}$ takes the values from a few to a dozen of $\mu$eV for the considered values of electric fields and grows by approximately 3 $\mu$eV per 1Vnm$^{-1}$. In the conduction band (Fig. 3b) the corresponding values are almost ten times bigger than in the valence band, and vary between 20 $\mu$eV to 100 $\mu$eV, for $E=1$ Vnm$^{-1}$ and $E=4$ Vnm$^{-1}$ respectively, with a linear growth of 33 $\mu$eV per 1 Vnm$^{-1}$. Similarly to $b^2$, the slope of $\Delta _{so}$ in the valence band changes slightly when the Fermi level $E_F\approx 32$ meV and the second valence band maximum (between the $\Gamma$ and *M* points in Fig. 1) enters the Fermi contour.

**Figure 4:**
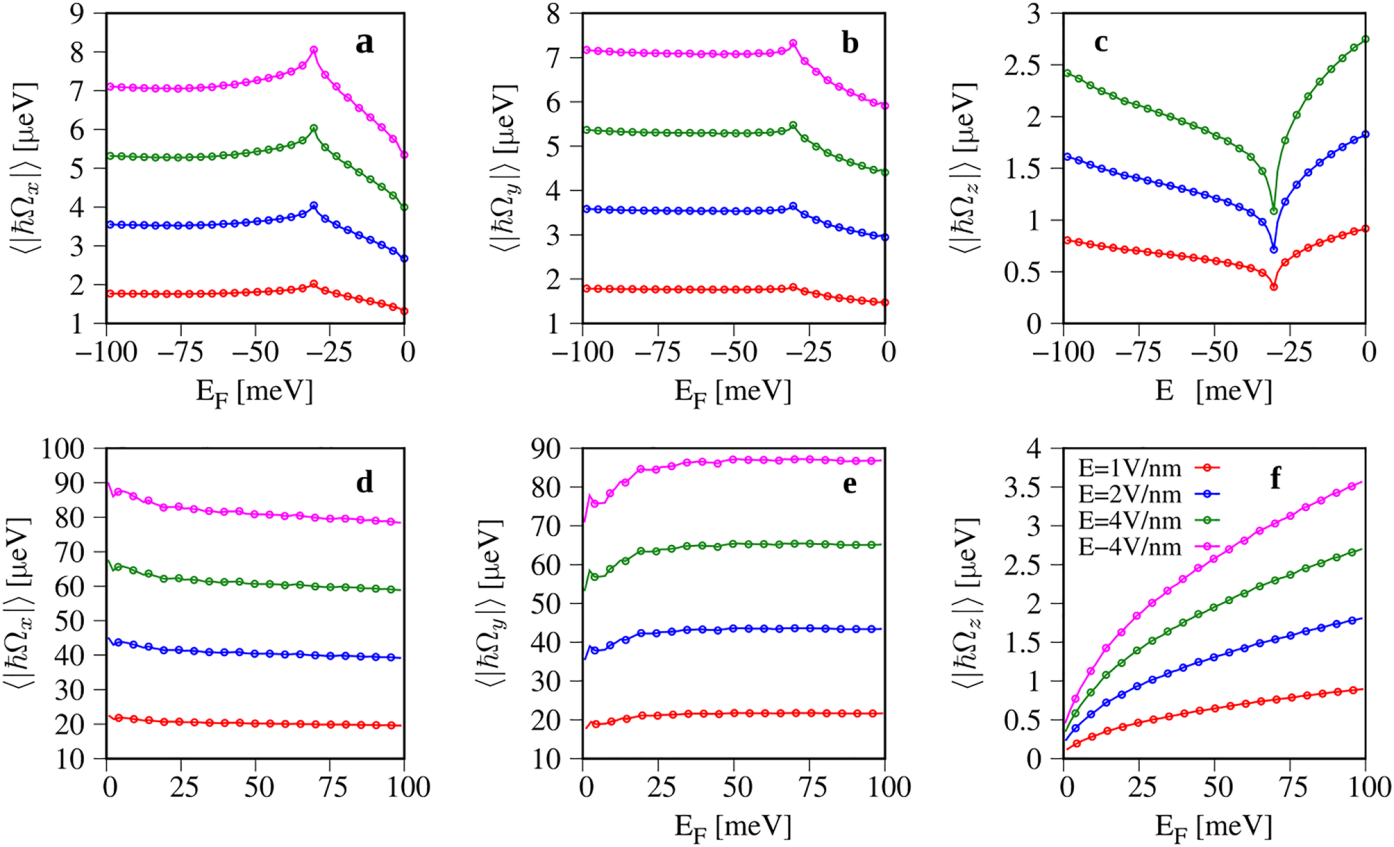
Fermi contour averaged components of the spin–orbit field $\hbar \Omega$ versus the position of Fermi level $E_F$ and for several values of electric field *E*. Panels (**a**–**c**) show the *x*, *y*, and *z* components of $\pmb {\Omega }$ for the valence band respectively; (**d**–**f**), same as (**a**–**c**) but for the conduction band. The Fermi energy is measured with respect to the valence (conduction) band maximum (minimum).

The spin splitting $\Delta _{so}$ characterizes the strength of the extrinsic SOC in a band. The anisotropy of SOC can be accessed through the components of the spin–orbit field $\pmb {\Omega }_{ k }$, $\Omega _{ k ,i}$ ,

6 $$\begin{aligned} \Omega _{ k ,i} = \frac{\Delta _{so}( k )}{\hbar } \frac{S_{ k ,i}}{S_{ k }} , \end{aligned}$$

where $S_{i},\,i = \lbrace x,y,z\rbrace$ is the expectation value of spin one-half operator at a given *k* point, and $S_k=\sqrt{S_{k,x}^2+S_{k,y}^2+S_{k,z}^2}$^39^. The calculated Fermi surface averaged components $\Omega _{i}$ are presented in Fig. 4. In the valence band (Fig. 4a,b), $\Omega _x$ and $\Omega _y$ take similar values, while $\Omega _z$ (Fig. 4c) is almost twice smaller and displays different monotonicity. In the conduction band, the in-plane spin components $\Omega _x$, $\Omega _y$ (Fig. 4d,e) are large, tens of $\mu$eV, and are doping independent, while $\Omega _z$ (Fig. 4f) takes the values of a few $\mu$eV, and is doping dependent. Such big differences between $\Omega _{x/y}$ and $\Omega _z$ lead to a sizeable, up to $\Omega _{x/y}/\Omega _z \approx 40$, doping-dependent anisotropy.

**Figure 5:**
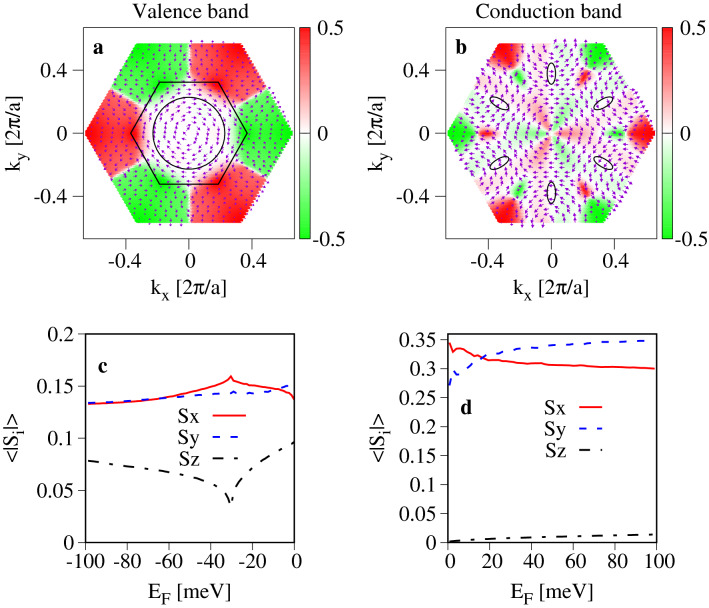
Effects of the extrinsic SOC on the electron spin: (**a**) the induced in-plane spin texture (arrows) and $s_z$ component of spin (color) for the top-most valence band and the electric field $E=1$ Vnm; (**c**) The corresponding Fermi surface averaged spin expectation values versus doping. The BZ wedge corresponding to doping in the range 0–100 meV is given by the area limited by the black hexagon and circle; (**b**,**d**) same as (**a**,**c**) but for the conduction band.

To understand these results one needs to look at the components of the electron spin which shape $\Omega _i$. We show them in Fig. 5a,b. The in-plane spin components $S_x$ and $S_y$ form the typical circulating Rashba spin texture (Fig. 5a). Within the considered doping range the length of $S_x$ and $S_y$ is approximately constant, as shown in Fig. 5c. The $S_z$ component displays much bigger diversity, and takes small values in the center of FBZ, while for bigger crystal momenta we observe the spin-valley locking effect with a strong spin polarization. In effect, when doping increases the average value of $|S_z|$ in the valence band first decreases from the value $|S_z| \approx 0.1$ to $|S_z| \approx 0.03$, and for $E_F \le -32$ meV its starts increasing and saturates at the value $|S_z| \approx 0.075$ (Fig. 5c). The qualitative change to $S_z$ takes place when the Fermi contour reaches the *k*-points close to the anticrossings marked by the green rectangle in Fig. 1a, what happens exactly at $E_F \approx -32$ meV. For *k* in the range from the $\Gamma$ point to the anticrossing at $E-E_F \approx -10$ eV, $S_z \approx 0$ (see the Supplementary Fig. S3). Increasing *k* towards the *K*-point, $S_z$ starts growing and reaches the maximum $S_z\approx 0.5$ at *k* above the anticrossing lying at $E-E_F \approx -9$ eV. Since the valence band maximum lies in between of the two anticrossings, we first observe a decrease of $|S_z|$ followed by its increase.

In the conduction band (Fig. 5b,d), the in-plane spins also form the Rashba texture, similar to the valence band, but the *z* component of spin is very small in the wedge of the BZ corresponding to doping range (represented by black ellipses). In effect, $\Omega _x$ and $\Omega _y$ take the values close to $\Delta _{so}$, while $\Omega _z$ is of the order of a few $\mu$eV (Fig. 4d–f), generating a large anisotropy of the extrinsic SOC.

As can be seen from the results shown above, SOC in nitrogene is weak. The calculated spin mixing parameter, $b^2 \approx 10^{-6}$, is approximately ten times bigger than for graphene^22^. This suggests, that the intrinsic SOC should not contribute much to spin scattering in nitrogene. Taking the typical momentum lifetime $\tau _p=100$ fs, we can make a rough estimate of spin lifetime from the Elliott-Yafet mechanism^29,31^,$\tau _s^{EY}\approx \tau _p/4b^2 \approx 50$ ns. On the other hand, several factors may affect SOC and spin dynamics in a material. For instance in graphene, the out-of-plane lattice distortions strongly affect both, the strength of the SOC and momentum scattering, dramatically reducing spin lifetime^40–43^. A transition from the flat to rippled graphene results in the emergence of an additional (intrinsic) SO term $\hbox {H}_{\text {curv}}$^42,44^. For typical ripples of radii in the range $R\sim$ 50 nm-100 nm the characteristic energy of this interaction is $\Delta _{\text {curv}}\approx 0.2$ K, and can exceed the energy of the intrinsic SOC, $\Delta _{\text {int}}\approx 0.01\,\text {K}-0.1$ K^23,42^, making the relevant spin-flip processes important. Additionally, rippling breaks the mirror symmetry ($z \rightarrow -z$) protecting the electron spin, and enables additional spin relaxation channels by flexural phonons and random spin–orbit fields^41,43^.

In contrast to graphene, nitrogene is naturally buckled. Thus, the effects of buckling are embedded in the intrinsic SOC and $b^2$, and are much stronger than effects of rippling discussed above. It was shown, that $\Delta _{\text {int}}$ at the K-point in graphene grows quadratically with the buckling height $\delta$, $\Delta _{\text {int}} \sim (\delta /a)^2$, *a* being the lattice constant, and for $\delta /a\approx 0.08$ it jumps to $\Delta _{\text {int}}\approx 1$ K^23^. In the case of nitrogene, the buckling height is $\delta \approx 0.9$Å, $a=2.3$ Å, which gives $\delta /a \approx 0.4$. Following the quadratic dependence for graphene, one gets for nitrogene $\Delta _{\text {int}}=25$ K, close to the value $\Delta _{so}^{K}=23$ K extracted from our first principles calculations. For comparison, static ripples of radii $R\sim$ 50 nm-100 nm, give the correction to the intrinsic SOC of the order of $\Delta _\text {curv}\sim a/R \approx 0.2$ K^42^,—negligible in the case of nitrogene.

Although lattice ripples should not significantly affect the intrinsic SOC in nitrogene, they can generate a small random spin–orbit fields leading to faster spin decoherence, in a similar way as it takes place graphene^41^. Fortunately, lattice rippling can be to a large extent eliminated by encapsulation of the host layer by, e.g., hexagonal boron nitride^45,46^.

spin–orbit coupling plays an important role in contemporary solid state physics, spintronics and topological quantum computing. Apart from these areas of physics, it has also been intensively studied in cold atoms systems. Therefore, in the next few lines we briefly compare SOC in crystalline solids with SOC in Bose gases.

In crystalline solids, SOC originates from the crystal potential, in which an electron is moving. In the rest frame of the electron, the electric field induced by the crystal potential acts as an effective, momentum dependent Zeeman field acting on the electron spin. The strength of SOC in a band is determined by the chemical composition of the crystal (materials made of heavier elements display stronger SOC effects than those made of light elements) and the topology of the band structure, and to some extent may be modified externally, e.g., by electric fields^47^, strain^48^, proximity effects^49^ or twisting^50^. The form of the spin–orbit interaction is dictated by the symmetry of the crystal; for instance, the famous Bychkov–Rashba^28^ and Dresselhaus^51^ types of SOC result from broken structure and bulk inversion symmetry, respectively.

In cold bosons systems, SOC is realized by coupling the motion of an atom to its internal (hyperfine) pesudo-spin states, corresponding to the electron’s spin *up* and spin *down* states^52–56^. Implementation of such coupling, called *synthetic* SOC^54^, requires an extra effort of dressing atomic states with lasers, but offers a full control of this interaction at will^57^. By a proper combination of laser fields and atomic pseudo-spin states, a variety of SOCs can be created and dynamically modified^54,57,58^. For example, Lin et al.^54^, realized one dimensional SOC, corresponding to equal contributions of Bychkov–Rashba and Dresselhaus SOC in conventional systems; Wu et al.^57^ implemented a scheme allowing for a controllable transition from 1D to 2D SOC. More exotic forms of SOC having no counterparts in real materials, such as, a 3D analogue of Rashba SOC^59,60^, are also possible, making cold atoms systems a powerful platform for exploring spin–orbit coupling and many body physics. Like in conventional materials, in bose gases, SOC is essential to the emergence of fascinating physical phenomena, e.g., a degenerate ground state of spin–orbit coupled Bose–Einstein condensates^61^, spatial separation of BEC^54,62,63^, quantum phase transitions^64^, or the existence of topologically non-trivial phases^57,60^. These few examples are a small sample of SOC-induced phenomena in cold atoms systems. A more detailed discussion of this topic can be found in Refs.^65–69^.

## Conclusions

We have investigated the fundamental spin–orbit coupling in buckled monolayer nitrogene. Based on first principles calculations we found that spin mixing parameter characterising the intrinsic SOC is small, on the order of $b^2\approx 10^{-6}$, and displays weak anisotropy. The extrinsic SOC, characterized by the Rashba spin–orbit fields $\Omega$, is also weak, on the order of $\mu$eV in the valence and tens to a hundred of $\mu$eV in the conduction band. Similar to the intrinsic SOC, the extrinsic SOC is also anisotropic. The anisotropy is particularly strong in the conduction band. Weak spin–orbit coupling in nitrogene suggests, that doped nitrogene or nitrogene nanoribbons may be attractive materials for spintronics applications.

## Methods

First-principles calculations were performed with the Quantum Espresso package^70,71^. The norm-conserving pseudopotential with the Perdew–Burke–Ernzerhof (PBE)^72,73^ version of the generalized gradient approximation (GGA) exchange-correlation functional was chosen, taking the kinetic energy cutoff of the plane-wave basis 80 Ry for the wave function and 320 Ry for charge density respectively. These parameters were found to give well converged results. Calculations with a hybrid functional were done with the Heyd–Scuseria–Ernzerhof (HSE06)^38^ functional, with the Fock exchange contribution 20%. Monolayer nitrogene was placed in a vacuum of 21 Å  to minimize interactions between periodic copies of the system. Self-consistency was achieved with $21 k_{x} \times 21 k_{y} \times 1 k_{z}$ Monkhorst-Pack grid^74^. The optimized lattice constant *a* was determined by minimizing the total energy followed by fitting a parabolic function. In each step the positions of atoms were fully relaxed by the quasi-Newton scheme as implemented in Quantum Espresso, assuming force convergence threshold $10^{-4}$  Ry/bohr. We found $a=2.29$ Å  and the out of plane buckling of the lattice $\delta =0.96$ Å, similar to those reported by other authors^13^. Calculations with the electric field were carried out with the dipole correction^75^. Fermi contour averages of spin mixing parameter $b^{2}$, spin–orbit field $\Omega ^{2}$ and spin components $s_{i}$ were calculated according to the formula

7 $$\begin{aligned} \langle A\rangle = \dfrac{1}{\rho (E_{F})S_{BZ}} \int _{FC} \dfrac{A_{k}}{\hbar |{\varvec{v}}_{F}(k)|}dk, \end{aligned}$$

where $A_{k}$ stands for $b_{k}^2$, $\Omega _{k}^{2}$ and $s_{k,i}$, $S_{BZ}$ is the area of the Fermi surface, $\rho (E_{F})$ is the density of states per spin at the Fermi level, ${\varvec{v}}_{F}(k)$ is the Fermi velocity.

## Supplementary Information

Supplementary Information.
