# Supplementary material for: Spin–orbit coupling in buckled monolayer nitrogene
Source: Sci Rep. 2022 Feb 25;12:3201. doi: 10.1038/s41598-022-07215-2 (PMC8881460; doi:10.1038/s41598-022-07215-2)
Supplement: Supplementary file 1 — Supplementary Information. [file 41598_2022_7215_MOESM1_ESM.pdf]

Supplementary Information: Spin-orbit coupling in buckled monolayer nitrogene.

Paulina Jureczko[1] **and Marcin Kurpas**[1,*]

[1]Institute of Physics, University of Silesia in Katowice, 41-500 Chorzów, Poland
[*]marcin.kurpas@us.edu.pl

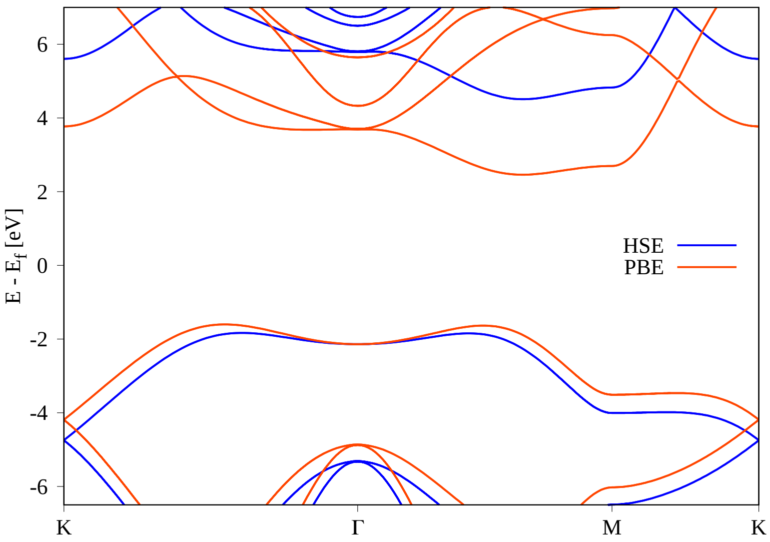

Figure S1. First principles band structure of nitrogene calculated with the HSE (blue) and PBE (red) exchange-correlation functionals.

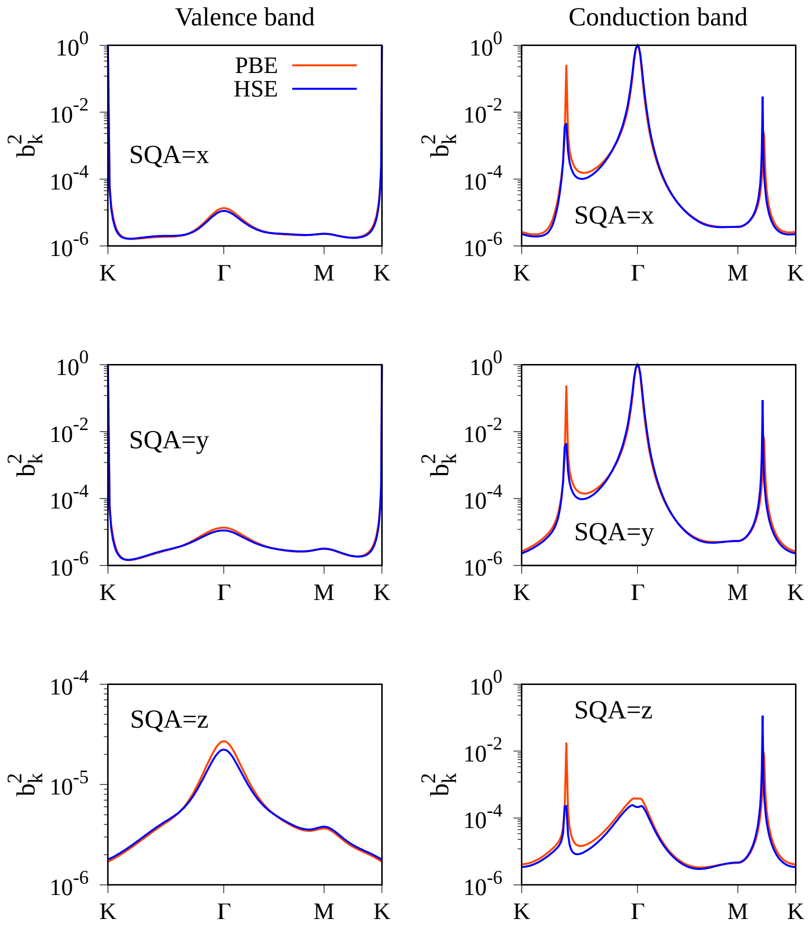

Figure S2. Spin mixing parameter b^2 ploted along high symmetry lines of the First Brillouin Zone for PBE (red) and HSE (blue) exchange-correlation functionals.

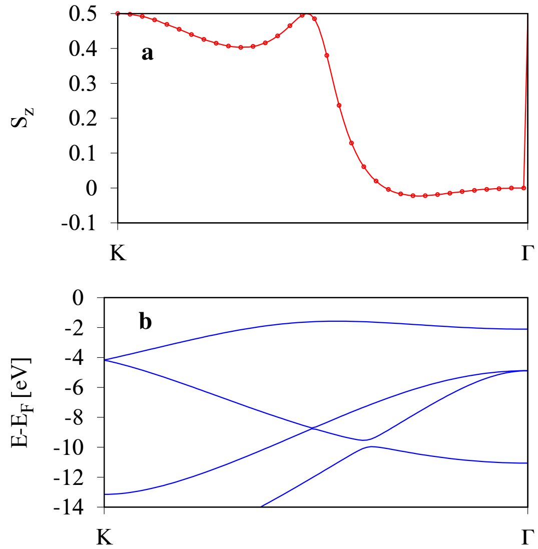

Figure S3. Expectation value of the z component of spin for the top-most valence band and electric field $E = 1$ E/Vnm-1 plotted along the $K\Gamma$ path of in the FBZ (a); (b) the corresponding band structure with only relevant valence bands shown.
